# Case of Poisoning by a Bee-Sting

**Published:** 1867-08

**Authors:** Wm. O’Daniel

**Affiliations:** Marion, Ga.


### ARTICLE V.

*Case of Poisoning by a Bee-sting.* By Wm. O'Daniel, M. D., of Marion, Ga.

On the 17th of May last, at 10 o'clock A. M., I was called to see Mr. B., a gentleman 32 years old, of feeble constitution. Upon my arrival, I was informed by his wife, that, about half an hour previously, he had been stung by a bee, on the right ear (helix). The sting was immediately removed, after which patient was troubled with nausea and occasional emesis, which continued to grow worse. After giving patient a careful and thorough examination, I found his condition to be as follows: Irrational, heart's action much enfeebled, breathing *exceedingly* difficult and stertorous, profuse flow of saliva, face considerably swollen, body partially covered with dark spots—more especially the face and neck; extremities cold.

I made use of the following: Sinapisms to extremities, morphia ¼ gr., brandy and water *pro re nata.* In one hour patient could speak rationally, and complained of a most excruciating pain in the epigastric region, chilly sensation, burning in fauces, and incessant nausea.

Mr. B. states that he has been stung by bees frequently during his life, and never suffered any unusual pain until about a year ago, when he was stung on the left orbital ridge, which produced very alarming effects: he was again stung on the end of the little finger, about three months ago, which caused much more intense suffering than before. The symptoms were similar in each instance.

By 4 o'clock P. M., Mr. B. was up, though much exhausted. Circulation 80 per minute. The point of injury presented no very unnatural appearance: it was red and a little swollen.

